# Cluster-randomised trial to test the effect of a behaviour change intervention on toilet use in rural India: results and methodological considerations

**DOI:** 10.1186/s12889-020-09501-y

**Published:** 2020-09-11

**Authors:** Wolf-Peter Schmidt, Kavita Chauhan, Priya Bhavsar, Sandul Yasobant, Vaibhav Patwardhan, Robert Aunger, Dileep Mavalankar, Deepak Saxena, Val Curtis

**Affiliations:** 1 Environmental Health Group, Department of Disease Control, London School of Hygiene and Tropical Medicine, Keppel Street, London, WC1E 7HT UK; 2 Indian Institute of Public Health Gandhinagar, Opp. Airforce Head Quarters, Nr. Lekawada Bus Stop, Chiloda Road, Lekawada CRPF P.O, Gandhinagar, Gujarat 382042 India; 3 Center for Development Research (ZEF), Bonn, Germany

**Keywords:** Sanitation, Behaviour change, Bias

## Abstract

**Background:**

Effective and scalable behaviour change interventions to increase use of existing toilets in low income settings are under debate. We tested the effect of a novel intervention, the ‘5 Star Toilet’ campaign, on toilet use among households owning a toilet in a rural setting in the Indian state of Gujarat.

**Methods:**

The intervention included innovative and digitally enabled campaign components delivered over 2 days, promoting the upgrading of existing toilets to achieve use by all household members. The intervention was tested in a cluster randomised trial in 94 villages (47 intervention and 47 control). The primary outcome was the proportion of households with use of toilets by all household members, measured through self- or proxy-reported toilet use. We applied a separate questionnaire tool that masked open defecation questions as a physical activity study, and excluded households surveyed at baseline from the post-intervention survey. We calculated prevalence differences using linear regression with generalised estimating equations.

**Results:**

The primary study outcome was assessed in 2483 households (1275 intervention and 1208 control). Exposure to the intervention was low. Post-intervention, toilet use was 83.8% in the control and 90.0% in the intervention arm (unadjusted difference + 6.3%, 95%CI 1.1, 11.4, adjusted difference + 5.0%, 95%CI -0.1, 10.1. The physical activity questionnaire was done in 4736 individuals (2483 intervention and 2253 control), and found no evidence for an effect (toilet use 80.7% vs 82.2%, difference + 1.7%, 95%CI -3.2, 6.7). In the intervention arm, toilet use measured with the main questionnaire was higher in those exposed to the campaign compared to the unexposed (+ 7.0%, 95%CI 2.2%, 11.7%), while there was no difference when measured with the physical activity questionnaire (+ 0.9%, 95%CI -3.7%, 5.5%). Process evaluation suggested that insufficient campaign intensity may have contributed to the low impact of the intervention.

**Conclusion:**

The study highlights the challenge in achieving high intervention intensity in settings where the proportion of the total population that are potential beneficiaries is small. Responder bias may be minimised by masking open defecation questions as a physical activity study. Over-reporting of toilet use may be further reduced by avoiding repeated surveys in the same households.

**Trial registration:**

The trial was registered on the RIDIE registry (RIDIE-STUDY-ID-5b8568ac80c30, 27-8-2018) and retrospectively on clinicaltrials.gov (NCT04526171, 30-8-2020).

## Background

Open defecation is thought to contribute to the transmission of gastro-intestinal infections especially in young children. The direct health benefits of high coverage with and use of toilets in rural areas have not been established and may be small [1, 2]. Toilet use may however be associated with indirect and non-health benefits such as reducing psycho-social stress in women [3].

India had, until recently, more than 60% of the global population that defecates in the open [4]. For more than three decades, the Government of India has made efforts to improve sanitation in rural India mainly by providing subsidy for toilet construction with some information, education and communication activities [5]. However, while coverage with toilets increased, there were often limited improvements in toilet use [5–8]. In 2014, through the launch of *Swachh Bharat Mission-Gramin* (SBM-G), the pace of toilet construction has increased [9]. Current strategies include a decentralised approach to improving sanitation coverage and use, by augmenting the capacity of state governments to undertake behaviour change activities and incentivizing government performance [10]. SBM-G focuses on mass media campaigns and village level events to address people’s toilet use behaviour [9]. Recent surveys show improvement in provision of toilets and toilet use [9]. However, the *Swachhta Status Report* as recent as 2015 [11] found that around 52% of the Indian population still defecated in the open. Similarly, a 2014 survey in rural villages in the north Indian states of Bihar, Madhya Pradesh, Rajasthan, and Uttar Pradesh found that 70% of the population defecated in the open. A resurvey in the same states in 2018 showed that this figure had reduced to 44% [12]. However, the same survey also found that 23% of people living in households with a toilet still defecated in the open. This figure contradicts findings from the National Annual Rural Sanitation Survey, which found that 97% of households in India with a toilet actually use it [13], perhaps highlighting methodological challenges in measuring toilet use in large surveys.

Hence, improving toilet use continues to be a public health challenge in India. Current efforts to change toilet use behaviour have often relied on traditional methods of health education [5, 7, 14], often making use of social pressure to effect behaviour change [12]. Creative, innovative and modern approaches to behaviour change, for example using models such as the Risks, Attitudes, Norms, Abilities, and Self-regulation framework (RANAS) [15] or Behaviour Centred Design (BCD) [16] have shown some promising results for handwashing [17, 18], food hygiene [19] and water treatment [15], but have rarely been applied to sanitation behaviour and have not been tested within a larger programme. Therefore, the present study aimed to test the effect of the 5-Star Toilet campaign, a scalable intervention which was developed using BCD, on the use of toilets in rural villages in the Indian state of Gujarat in the context of the ongoing SBM-Gramin programme. Toilet construction was not part of this intervention. The study was part of a comparative programme involving three other studies pursuing similar study designs, tools and behaviour change objectives. The other three trials were conducted by other groups in the Indian states of Odisha, Bihar and Karnataka, testing different behaviour change interventions to increase toilet use in households with an existing toilet [20].

## Methods

### Study design and setting

**Fig. 1:**
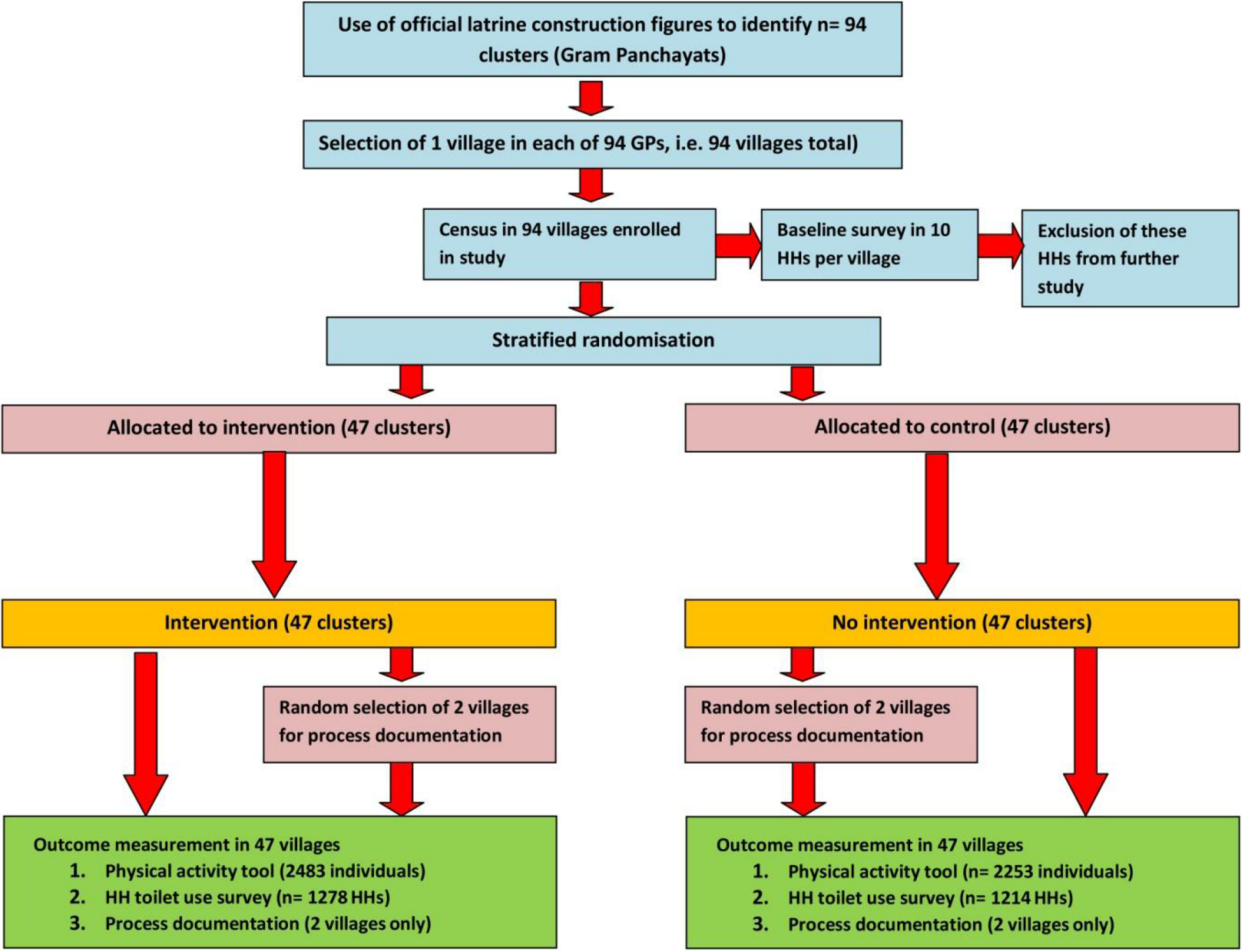
Study flow diagram

The study was a cluster-randomised trial with 47 intervention and 47 control clusters, conducted in the district of Bhavnagar, Gujarat, India. Bhavnagar was chosen because it was the last district in Gujarat to be declared open defecation free, and therefore deemed to have a high prevalence of non-use of toilets. The study design is depicted in Fig. 1. Overall, 94 villages were randomised to intervention or control groups. Outcomes (use of toilets by all household members and a range of secondary outcomes) were assessed in a cross sectional questionnaire survey post-intervention.

### Enrolment of villages

To enrol villages we compiled a list of all Gram Panchayats (an administrative unit in India consisting of one or more villages) in three blocks (Mahuva, Talaja and Palitana). There were a total of 335 villages from 325 Gram Panchayats. For our study, Gram Panchayats were eligible for enrolment if, based on government records, they had at least 70% toilet coverage (*n* = 137). When there were multiple villages within a Gram Panchayat, we selected the village with the higher reported coverage. From the list of 137 villages, 110 villages were selected using probability proportional to size sampling using data from the National Census of 2011. We conducted a household census in 106 of the 110 clusters (4 were excluded due to logistics). We excluded 16 villages with the lowest coverage, arriving at 94 villages selected for the study. Out of the 94 clusters, three clusters had populations of more than 300 households. We therefore used chunking to segment these larger villages into multiple parts and then selected two segments of approximately 150 households in each village which were both enrolled as the same cluster. Toilet coverage in the resulting villages was lower than expected prior to the census (mean 60.0%, range 9%–100%). Randomisation was done in 13 strata. We created 5 different strata of toilet coverage (0–24%, 25%–44%, 45%–59%, 60%–74%, 75%+) and 3 different strata of household tap water coverage at village level (0% to 49%, 50% to 74%, 75%+). We predicted that these two variables might correlate with toilet use and the success of the intervention. The combination of these two strata resulted in 13 different strata (stratum size ranging from 2 to 20 villages). A minimum 3 km distance was maintained between intervention and control clusters.

### Enrolment of households

We carried out a household census, and from this list randomly selected 40 eligible households per village for the study. Households were eligible if they had received any assistance, either monetary or any other, under any government programme, to construct a toilet and had a functional toilet, defined as having; 1) a pan that is not broken, and 2) a functional connection to a pit (single or twin pits).

From the list of 40 households per village we randomly allocated 10 households to the baseline survey and 30 to the follow up survey (post-intervention). The baseline study was done to estimate the overall baseline prevalence of the outcomes and the intra-class correlation coefficient to confirm the sample size calculation. The 10 baseline households per village were discarded from further study. In the other 30 households, toilet use and other detailed sanitation-related data were collected only post-intervention, not at baseline.

### Intervention

**Fig. 2:**
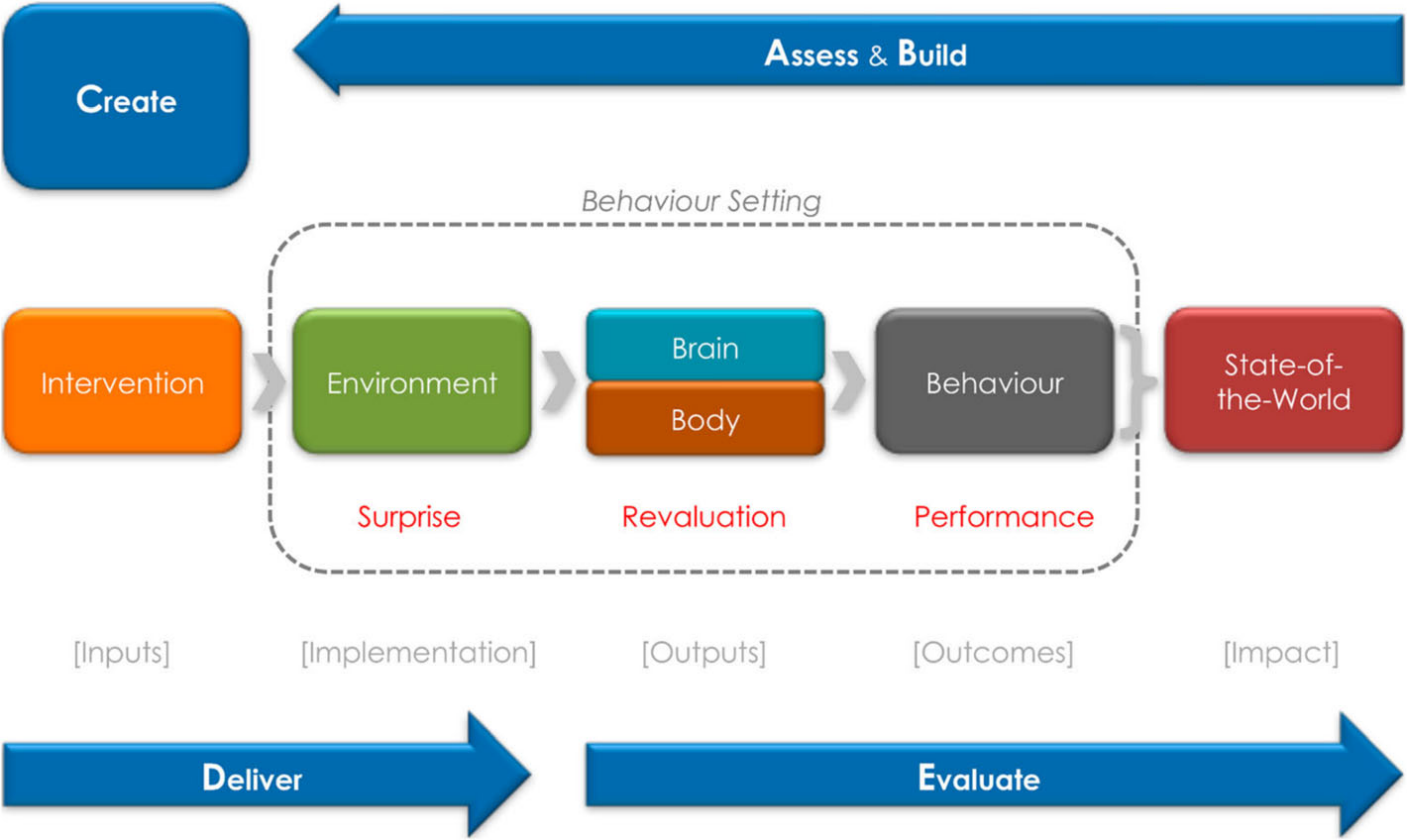
BCD concept, from [16]

The ‘5 Star Toilet’ campaign used the BCD framework and theory of change (ToC) to design the intervention [16] (Fig. 2). BCD’s theory of change involves five steps: *Assess*; (review what is known about the determinants and context of the target behaviour); *Build* (gather data from the field to understand the determinants of behaviour in-situ); *Create* (work with a creative team to identify a novel, surprising insight about the target behaviour, incorporated into intervention components); *Deliver* (implement the components of the intervention) and *Evaluate* (determine the effectiveness of the implementation vis a vis behaviour change). A detailed description of the intervention development has been published elsewhere [21]. The Assess step included a literature review and a Framing Workshop. Mixed methods formative research was conducted in villages close to the study villages that were not part of the actual trial to identify the determinants of toilet use/non-use in the study population and to arrive at a design brief (*Build*). Formative research methods included structured conversations (*n* = 40) with the help of a discussion guide, and range of other research tools [21]. We also carried out a survey in 200 households to understand toilet coverage and functionality. These insights were then organised using the BCD checklist of potential factors in the environment, physical setting and informants’ minds and included water availability, pit filling, knowledge about disease, manners, shame, dignity, safety, comfort, nurture, routine and habit. The creative process (*Create* step) involved brainstorming and reflection to generate concepts and ideas to address the determinants of toilet use. The ‘5 Star Toilet’ concept was nested within the campaign theme of ‘The World is Getting Smarter’ and a lifestyle is not ‘completely smart’ until people have a toilet that matches the quality of their other ‘smart’ belongings such as smart phones, laptops, gadgets etc. A general theme of the intervention was the attempt to position the toilet as a modern appliance, much like the mobile phones and bikes. This lead to the concept of presenting the intervention in a “digital context” by employing innovative methods such as “Virtual Reality” toilets, “Pi-Fi” (A local wifi where one can download campaign films without mobile data), and films shot in a village and showed back using a casting device, or uploaded on YouTube. Components of the intervention were piloted separately and as a package.

The final iteration of the intervention was rolled out by the Coastal Salinity Prevention Cell (a local NGO) from mid-September to December 2018. The intervention was delivered during 2 days in each village by two teams comprising three trained facilitators and performing artists (Table 1). Day 1 and Day 2 were separated by about 4 weeks. Facilitators had at least an undergraduate degree in social sciences or an equivalent qualification and were trained over a 3 week period prior to the piloting of the intervention.

**Table 1:** Campaign components

Activity	Description	Tools
**Pre intervention delivery**		
**Meetings to seek support**	Meetings were held with village leaders to discuss the campaign and get support to plan and organize day 1 event.	Facilitation script.
**Recruitment of volunteers**	1 volunteer identified from each village with help of local NGO partner/ *Sarpanch* who would promote 5 Star Toilet concept, help the team of facilitators to deliver the day 1 and day 2 intervention and follow up with community members for 5 Star Toilet makeovers.	Training of volunteers.
**3 Days before day 1 event**	Call/s were made to each village volunteer to ensure the WhatsApp broadcast groups are formed to share information on the campaign with the community members, mobile teasers have been passed around, leaders met with and locations identified for event/s.	WhatsApp teasers, phone calls
**DAY 1**		
**Announcements**	A customized campaign vehicle to go around the village to make announcements and also carry all the material for the events.	Vehicle design, announcement script, song recording, media player
**Interaction with volunteer**	Facilitators, with the help pf volunteer, identify location for the evening event and create a route plan for the household visits and street events.	
**Interaction with children**	Expose children to a virtual reality (VR) experience of a 5 star toilet design and the idea of 5 star toilets and teach them slogans around 5 star toilets.	VR App on 5 star toilet design, VR googles, phone
**Household Visits**	Two teams of facilitators + Artists + Van + Children go from street to street, making household visits. Expose the idea of 5 star toilets, enquire if they would like to know the rating for their toilets, rate their toilet and express appreciation for what they already have. If they have 5 star: Award them with a 5 star sticker and paste it on their toilet and invite them to the evening event to receive certification. Take photographs. If they don’t have 5 star: Explain what they need to do to get 5 star.	5 star toilet leaflet 5 star toilet poster
**Van in the community**	Park the van in the street and make announcements, play songs, display 3D photographs of different toilet innovations from around the world, display small toilet model, and VR experience of a 5 star toilet.	Music player, photographs, mobile, VR goggles, VR App
**Preparations for the evening event**	Set up the venue for the evening event: AV + seating arrangement for community members, download photographs of the day’s activities from phone/camera and write certificates for 5 star toilet awardees.	Certificates, AV system, rug for seating arrangement
**Enrolment Corner**	In parallel create an enrolment corner for households willing to improve their existing toilets into a 5 star toilet with a standee on 5 star toilets, a table to showcase 5 star toilet model and a toilet chair on display for differently abled people.	Leaflets and documentation sheet, toilet chair, smart network Wi-Fi
**Evening Event**	1. Play the campaign song and interact with the children and make announcements 2. Play Films – Saving Time and Saving Effort 3. Skit performance 4. World of Toilets (slide show) 5. Toilet makeover films and toilet chair films 6. Celebrate those with 5 star toilets/ 5 star+ by awarding certificates 7. Introduce those who have enrolled – call them to the front and celebrate them 8. Farewell – “All the best! We will come back in 2–3 weeks to celebrate again”.	AV equipment, films, artists, certificates
**Follow up**	Volunteers promote 5 star toilet makeovers between day 1 and day 2 events. Take photographs of families who have modified their existing toilets.	Home visits, follow up on phone
**Share films and song**	Share films and campaign song in the village WhatsApp groups.	WhatsApp, YouTube link https://www.youtube.com/channel/UCqmL6DxtcDpAKeIU4Io33ig
**DAY 2**		
**Organize**	All the pre-post toilet makeover photographs from the village are compiled into a presentation – clearly marking the names of people.	Laptop/Tablet
**Announcements**	Make announcements about the evening event.	Van, audio system, announcement script.
**Testimonial Videos**	Record videos of families that undertook 5 star toilet makeover.	Phone camera
**Evening community event**	Make preparations, identify site.	
**Guessing Contest Pit Filling**	Participants asked to guess how fast a pit fills up. This is done through a life size pit standee. The facilitator explains the time it takes for a pit to fill and explains the process of composting.	Live sized pit standee
**Guessing Contest Compost**	Jars with normal soil and compost are kept on a table. Participants are invited to guess which jar contains compost.	Glass jars with soil and compost.
**Films of pit filling and testimonial videos**	Films of pit filling are showcased and videos of people who undertook toilet makeovers are played.	Testimonial films, short films
**Toilet Board**	Photographs of people who did toilet makeover are displayed on a board and the board in placed in village centre or *Panchayat Gahr*.	Board, pictures, printer
**Toilet Makeover**	Presentation of certificates to those who improved toilets. Invite participants to come and share their experience with those in attendance.	Pre/post presentation.
**Farewell**	People are thanked for their participation.	

### Outcomes and outcome assessment

The primary outcome of toilet use was defined as the proportion of households where all members above the age of 5 years were reported to use the toilet the last time they defecated. This outcome was measured using a questionnaire which also covered secondary study outcomes, i.e. exposure to the intervention, perceptions around toilet ownership in the community, agreement with sanitation related statements, and directly observed toilet characteristics. Apart from exposure to the intervention, the questions asked were the same at baseline and endline. The survey was addressed to one adult respondent per household, preferably the male or female household head. A gap of 6 weeks was maintained between campaign roll out and endline data collection. The questionnaire was simultaneously administered in intervention and control clusters.

Because of concerns regarding over-reporting of toilet use, we developed an alternative tool to reduce socially desirable responses and responder bias. Specifically for the purposes of this study, we designed a questionnaire about physical activities relating to diet and incidental activities concerning the daily routine of respondents, with time spent for each physical activity measured in minutes (categorised as < 10 min, 10-30 min, > 30 min, or NOT doing this activity). Among the questions, there was one question about time spent to walk to the field for defaecation, with “using toilet at home” offered as a possible response. This questionnaire was delivered by a separate team from a different data collection company that was kept blind to the real purpose of the survey. The tool was administered about 1 week before the endline survey in each village to make it less likely that respondents would link the physical activity study to the intervention or the subsequent main endline survey. The questionnaire was completed by one or two people per household (preferably one female and one male) aged 18 and above who were present at the time of the visit and who were only asked about their own physical activity. If more than two eligible persons were found in a household, two individuals were selected at random. If only one eligible person was present in a household, the team visited an additional household until the target number of 60 interviews per village was met. The complete physical activity questionnaire is added as Supplementary material.

Both teams (main questionnaire team and physical activity team) were given the same list of about 30 households per village selected for the endline survey at baseline (see above). To account for non-availability of households due to migration, refusal to take part in the survey and other factors, we randomly selected an additional 15 households per cluster, or fewer, depending on availability. Both data collection teams selected from this additional list households to replace households that were not available, or (for the physical activity questionnaire) if only one eligible person was found. As the two teams for the physical activity tool and the endline toilet use questionnaire worked independently, only 66% of final household samples overlapped (Fig. 1).

### Process evaluation

The process evaluation aimed to understand the reasons for the results of the 5 Star Toilet Campaign. Data collection methods and sources used to assess the process included; 1) Document review (reports, newspaper clippings, and government BCC strategy paper), 2) Field observations (*n* = 6) and review of activity logs to assess intervention fidelity, and participation of community, 3) Semi-structured interviews (*n* = 14) with SBM officials, the design team, intervention delivery team, and participants from intervention and non-recipients from control clusters, 4) Focus Group Discussions (*n* = 5) with programme staff (*n* = 1) and participants (2 each with women and men, 8–10 participants per group) to explore views on the campaign and the perspectives on toilet use and non-use. In depth process evaluation was done in 2 intervention and 2 control clusters chosen at random (Fig. 1). Results have partially been published elsewhere [21] and will be reported in full in a future paper.

### Sample size

We assumed that in 65% of households with a government supported toilet, all household members aged 5 years or older would be using this latrine. This figure was based on formative research in the study area that found that about 44% of households had members who go for open defecation. We assumed that use would increase to 75% (by 10% points) compared to the control arm, which was deemed an effect size of public health interest. Using a standard formula to calculate a sample size for the comparison of two proportions resulted in requiring 349 households per arm to detect this 10% difference with 80% power and an alpha of 0.05, ignoring village level randomisation. We assumed an intra-class correlation coefficient (ICC) of 0.1 based on a sanitation trial in Orissa [22]. We chose to enrol 30 households per village cluster, as enrolling more only marginally reduced the number of required clusters. This resulted in 45 villages per arm, and 30 households per village at a design effect of 3.9. This figure was increased to 47 villages per arm to account for loss to follow-up of households and whole clusters.

### Statistical analysis

Prevalence differences across trial arms were calculated using linear regression (function: Gaussian, link: identity). Clustering at village level was adjusted for by using generalised estimating equations and robust standard errors. The primary endpoint analyses were done using unadjusted models (intention-to-treat analysis). Due to some imbalance in socio-economic characteristics, all analyses were finally adjusted for an asset index (continuous variable) and maximum male education level (dichotomised into primary schooling or less vs secondary or higher). The asset index was constructed using principal component analysis of 9 socio-economic variables. The first predicted component explained 27.7% of variation and was chosen as asset index (KMO: 0.73). Sample size calculations and all analyses were done in STATA 14 (StataCorp).

The trial was prospectively registered on the RIDIE registry (RIDIE-STUDY-ID-5b8568ac80c30, 27-8-2018) and retrospectively registered on clinicaltrials.gov (NCT04526171, 30-8-2020).

## Results

Of the 1384 intervention households selected for the main questionnaire, 351 (25.3%) could not be found or did not in fact, have a latrine (were ineligible), and 26 (1.9%) did not consent. Two hundred seventy-one households were added from the list in random order, resulting in 1278 households (6679 individuals) enrolled for the endline survey. Of the 1333 control households selected for the endline survey prior to the intervention, 331 (24.8%) could not be found or did not in fact have a latrine (were ineligible), and 33 (2.5%) did not consent. Two hundred forty-five households were added from the list in random order, resulting in 1214 households (6174 individuals) enrolled for the endline survey. The physical activity questionnaire was done in 4741 individuals (2483 intervention and 2253 control participants), of which 3114 (66%) were from households also included in the main questionnaire.

Table 2 shows the socio-economic characteristics of control and intervention study populations by intervention arm from the endline survey. Good balance was achieved with respect to household size, caste, religion, female education and house structure. Some imbalances were observed, with male education and graduate level education being more common in the intervention arm. There was also some imbalance in the distribution of the asset index, with intervention households more commonly found in higher asset quartiles.

**Table 2:** Socio-demographic and socio-economic characteristics of households at endline enrolled for follow up study

Item	Control		Intervention		Prevalence difference, %
	N	%	N	%	
**Total**	1214		1278		
**Household size**					
1–3	252	20.8	267	20.9	0.1
4–5	398	32.8	436	34.1	1.2
6–7	365	30.1	324	25.4	−4.5
8+	199	16.4	251	19.6	3.1
**Caste**					
SC/ST	56	4.6	39	3.1	−2.3
OBC	730	60.1	792	62.0	1.7
General	315	26.0	357	27.9	2.9
Prefer not to disclose	113	9.3	90	7.0	−1.8
**Religion**					
Hindu	1200	98.9	1264	98.9	0.1
Muslim	14	1.2	14	1.1	−0.1
**Highest female education level (*n*** **= 2467)**					
No formal schooling	277	23.0	259	20.4	−2.5
Primary	161	13.4	181	14.3	1.1
Secondary	682	56.6	716	56.4	−0.3
Diploma	9	0.8	10	0.8	0.1
graduate	77	6.4	104	8.2	1.7
**Highest male education level (*n*** **= 2449)**					
No formal schooling	83	6.9	82	6.5	−0.3
Primary	156	13.0	160	12.7	0.1
Secondary	774	64.7	727	57.7	−7.0
Diploma	29	2.4	23	1.8	−0.5
graduate	156	13.0	269	21.3	8.6
**Asset index quartile**					
Lowest	348	28.7	294	23.0	−5.6
Low intermediate	319	26.3	288	22.5	−4.1
High intermediate	295	24.3	340	26.6	2.6
Highest	252	20.8	356	27.9	7.4
**House structure**					
Kutcha	158	13.0	165	12.9	−0.1
Semi-pukka	619	51.0	631	49.4	−1.5
Pukka	437	36.0	482	37.7	1.7

Table 3 shows the effect of the intervention on the primary study outcomes (three responses in the intervention arm and 6 responses in the control arm were missing). At baseline reported toilet use by all household members was 87.0% of households in the control and 83.4% of households in the intervention arm. At follow up reported use of the toilet by all household members was 83.8% of households in the control group and 90.0% in the intervention group, i.e. 6.3 percentage points (95%CI 1.1, 11.4) higher in the intervention compared to the control arm. This effect size was slightly attenuated after adjusting for asset index (as continuous variable) and highest male education in a household (dichotomised into illiterate to primary vs secondary or higher) to + 5.0% (95% CI -0.1, 10.1). A similar effect size was found for the analysis of the prevalence of toilet use in individual household members (not collapsed at household level) (85.1% vs 91.2%, adjusted difference + 4.6% difference, 95% CI 0.5, 9.7). Overall, toilet use by all household members in the control arm at baseline (87%) was similar to toilet use by all household members observed in the control arm at follow up (84%), suggesting an absence of a temporal trend from baseline to follow up, or an absence of an effect of the trial procedures on the reporting of behaviour.

**Table 3:** Effect of the intervention on study outcomes

Item	Control		Intervention		PD, %	95% CI	APD%	95% CI	ICC
	N	%	N	%					
**Baseline**									
Use of toilet by all household members (irrespective of apparent toilet use)	328	87.0	303	83.4	−4.9	–			
**Endline**									
*Primary outcome*									
Use of toilet by all household members	1208	83.8	1275	90.0	6.3	1.1 / 11.4	5.0	−0.1 / 10.1	0.14
*Secondary outcomes*									
Individually reported toilet use (reported use not collapsed at household level)	6174	85.1	6679	91.2	6.1	1.1 / 11.2	4.6	−0.5 / 9.7	0.17
Individually reported toilet use (physical activity tool)	2253	80.7	2483	82.2	1.5	−3.4 / 6.4	–	–	0.12
Individually reported toilet use (physical activity tool) restricted to households also taking part in endline survey	1636	82.8	1736	85.9	3.3	−1.7 / 8.3	1.7	−3.2 / 6.7	0.11

*PD* prevalence difference, calculated using linear regression (function: Gaussian, link: identity). Clustering at village level was adjusted for by using generalised estimating equations and robust standard errors. *APD* adjusted prevalence difference. PD was adjusted for asset index (continuous variable) and maximum male education level (dichotomised into primary or less vs secondary or higher)

The physical activity questionnaire produced a 4.4% point lower overall estimate of individual toilet use than the endline tool (84.5% vs 88.9%). No major effects of the intervention on toilet use were observed, with or without adjusting for asset index and male education (difference + 1.7%, 95%CI -3.2, 6.7). The estimates were not greatly affected by restricting the analysis to households also part of the main survey. For the adjusted analysis, the test for interaction to explore the difference in effect estimates between the main questionnaire tool (effect + 5.0%) and the physical activity tool (effect + 1.7%) showed a *p*-value of 0.09, suggesting some evidence for a difference in effect estimates.

As shown in Table 4 the intervention had only a limited effect on observed toilet characteristics. Minor effects were found including a 6.4% point increase for availability of a water container, slippers and cleaning materials, as well as in 4 of the 5 attributes promoted by the 5 Star Toilet campaign (painted walls, cleanliness, light bulb, water available), but the confidence intervals were wide, while the effect sizes were reduced after adjusting for asset index and male education. Slightly more toilets in the intervention arm than in the control arm were found to be in apparent use.

**Table 4:** Effect of intervention on observed toilet characteristics

Item	Control		Intervention		PD, %	95% CI	APD%	95% CI
	N	%	N	%				
**Latrine use for other purpose**	1214	9.6	1278	6.3	−3.3	−6.4/− 0.2	−2.6	−5.6/ -0.4
**Clogging of squatting pan**	1214	15.0	1278	10.6	−4.2	−8.4/0.0	−3.2	−7.4/ 1.0
**Availability of water container**	1214	84.9	1278	89.1	4.2	−0.7/9.0	3.3	`-1.6/ 8.1
**Availability of slippers**	1214	19.8	1278	24.9	4.8	0.1/ 9.4	3.0	−1.8/ 7.7
**Availability of cleaning materials**	1214	77.6	1278	84.3	6.4	0.8/ 12.0	5.0	−0.7/ 10.6
**Toilet is in apparent use**	1214	86.1	1278	90.4	4.3	−0.6/ 9.2	3.1	−1.8/ 8.0
**Made any changes in last 6 months**	1214	6.3	1278	6.0	−0.2	−2.4/ 1.9	− 0.3	−2.5/ 1.9
**Plan to make any changes**	1214	27.6	1278	22.9	−4.7	−9.3/ 0	−3.6	−8.3/ 1.2
**Five star items**								
Painted walls	1214	44.9	1278	52.9	8.1	1.9/14.2	5.3	−0.8/11.5
Clean	1214	68.5	1278	76.0	7.4	1.3/13.4	5.7	−0.4/11.7
Light bulb	1214	53.4	1278	62.4	9.3	1.8/16.8	6.9	−0.3/14.1
Ventilation	1214	18.0	1278	18.8	0.8	−3.0/4.7	0.4	−3.6/4.3
Water	1214	39.0	1278	47.6	8.9	2.2/15.6	5.3	−1.0/11.7

*PD* prevalence difference, calculated using linear regression (function: Gaussian, link: identity). Clustering at village level was adjusted for by using generalised estimating equations and robust standard errors. *APD* adjusted prevalence difference. PD was adjusted for asset index (continuous variable) and maximum male education level (dichotomised into primary or less vs secondary or higher)

Compared to the control group, intervention households more often reported having heard of, or attended, community events on sanitation. They also reported higher exposure to most campaign-specific elements, such as pit filling demonstrations, using a chair for assisting the disabled in the toilet, or seeing a small model of a 5 star toilet (Table 5). A higher proportion of respondents in the intervention arm reported making changes to their toilets. However, overall campaign exposure was low. Only about 14% of intervention households had heard the term “5 Star Toilet” (3% in control). Four percent could show a certificate awarded by the campaign team (almost nobody in the control arm could). Only 18% of households in the intervention arm had seen the skit (5% in control arm), and 13% had seen the 5 Star Toilet model (2% in control arm). Exposure to most other campaign items showed an intervention-control difference of less than 10% points.

**Table 5:** Exposure to Intervention

Item	Control		Intervention		PD, %	95% CI	APD%	95% CI
	N	%	N	%				
**Recently heard about toilets in any of these contexts (in last 6 months)**								
Conversation with others	1214	6.7	1278	9.9	3.1	0.6/5.6	2.8	0.3/5.4
Visits to neighbours	1214	3.1	1278	4.5	1.3	−0.2/2.8	1.2	−0.3/2.8
WhatsApp message	1214	2.1	1278	3.1	1.0	−0.7/2.7	0.4	−1.1/2.0
Village meeting	1214	14.3	1278	23.5	9.1	5.1/13.1	8.4	4.3/12.4
Event in community	1214	13.1	1278	30.0	16.7	11.4/22	16.3	11/21.6
Posters /stickers	1214	6.9	1278	13.2	6.5	3.6/9.5	6.2	3.2/9.2
Radio	1214	0.4	1278	0.6	0.1	−0.4/0.7	0.0	−0.1/0.1
TV	1214	21.9	1278	22.9	1.3	−3.3/5.8	0.0	−0.5/4.4
**What did you hear**								
One should construct a toilet if a household doesn’t have one	1214	14.4	1278	19.9	5.6	2.0/9.2	5.2	1.6/8.9
One should improve one’s toilet if it is poor quality	1214	6.7	1278	12.1	5.4	3.1/7.7	5.2	2.8/7.5
One should use toilet for defecation instead of going out in the open	1214	18.5	1278	25.0	6.3	2.5/10.1	5.8	2.1/9.5
**After hearing this did you make changes to your toilet or done anything as a consequence?**								
talked with someone	1214	15.0	1278	18.3	3.4	−0.1/7.5	3.1	−1.1/7.3
made changes to my toilet	1214	8.0	1278	12.8	4.6	1.3/7.8	4.0	0.8/7.2
saved money for a toilet	1214	3.5	1278	3.6	−0.1	−1.8/1.8	0.2	−1.6/2.0
**Heard of any community event that talks about toilet in the past 6 months**	1214	18.5	1278	39.1	20.7	15.4/26.0	19.7	14.3/25.1
**Attended such an event**	1214	8.3	1278	22.3	13.9	10.6/17.1	13.3	9.9/16.7
**Promote toilet improvement**	1214	6.5	1278	18.3	11.7	8.8/14.6	11.2	8.1/14.2
**Commit to improve toilet**	1214	22.8	1278	12.3	−12.3	−21.2/−3.5	−12.5	−21.4/−3.5
**Heard the phrase ‘5 star toilet’**	1214	2.6	1278	13.9	11.3	8.9/13.8	10.9	8.5/13.4
**Where did you hear it**								
TV	1214	0.7	1278	1.6	0.1	0.0/1.9	0.1	0.0/1.9
Village meeting	1214	1.2	1278	5.2	3.9	2.6/5.2	0.4	2.4/5.1
Community event	1214	1.8	1278	10.9	9.1	6.7/11.5	8.8	6.4/11.2
WhatsApp message	1214	0.3	1278	0.5	0.1	−0.4/0.6	0.1	−0.4/0.1
Posters/stickers	1214	0.7	1278	4.1	3.4	1.9/5.0	3.2	1.8/4.7
Virtual Reality film	1214	0.4	1278	0.9	0.5	−0.2/1.3	0.5	−0.3/1.3
Friend/relative	1214	0.3	1278	0.7	0.4	−0.3/1.1	0.3	−0.4/1.1
**Certificate for a 5-star toilet**	1214	0.4	1278	4.5	4.0	0.3/5.1	3.8	2.7/4.8
**Picture of your family on the village ‘Toilet Board’ poster**	1214	0.2	1278	4.8	4.5	3.3/5.7	4.3	3.2/5.5
**Skit about toilet convenience**	1214	4.9	1278	18.2	13.1	10.0/16.3	12.6	9.4/15.8
**Seen small-sized 5-star toilet model**	1214	1.9	1278	12.5	10.7	8.2/13.1	10.3	7.8/12.8
**Certificate about your toilet, or know anyone who has**	1214	1.5	1278	7.4	5.9	4.2/7.6	5.7	3.9/7.5
**Seen a certificate give-away**	1214	2.0	1278	11.2	9.2	0.7/11.4	9.0	6.7/11.3
**Someone talking about or showing a movie about pit filling**	1214	2.4	1278	10.1	7.6	5.5/9.7	7.4	5.2/9.7
**Movie about using a chair in the toilet for disabled or elderly people**	1214	2.9	1278	11.0	8.0	6.0/10.1	7.8	5.6/10.1
**Use any of the following**								
Facebook	1214	18.2	1278	23.6	5.2	1.3/9.1	2.1	−1.6/5.7
WhatsApp	1214	24.0	1278	31.2	7.1	3.0/11.2	3.0	−0.6/6.7
Instagram	1214	6.0	1278	8.7	2.6	0.0/5.2	0.8	−1.5/3.1
YouTube	1214	19.2	1278	22.1	2.7	−1.4/6.8	−0.8	−4.5/2.9
**Ever got or sent a message on WhatsApp about toilets**	1214	2.3	1278	2.8	0.6	−0.7/1.9	−0.1	−1.3/1.2
**Heard about Swachh Sunder Shauchalay campaign**	1214	40.6	1278	45.8	5.3	0.6/10.1	3.7	−0.8/8.2
**Swachh Sunder Shauchalay campaign is about**								
Paint your toilet walls	1214	7.9	1278	8.8	0.9	−1.5/3.3	0.7	−1.8/3.1
Decorate your toilets	1214	28.1	1278	32.6	4.5	0.2/8.7	3.4	−0.8/7.6

*PD* prevalence difference, calculated using linear regression (function: Gaussian, link: identity). Clustering at village level was adjusted for by using generalised estimating equations and robust standard errors. *APD* adjusted prevalence difference. Pd was adjusted for asset index (continuous variable) and maximum male education level (dichotomised into primary or less vs secondary or higher)

There was little difference between intervention and control regarding agreement with statements reflecting important campaign messages such as “Toilets are not just for women; men should use them too”, “A smart person is one who uses a toilet”, or (phrased negatively) “Toilet pits fill quickly if too many people in the household use them” (Table 6). Consistent with the finding that slightly more intervention arm respondents reported improving their toilets, they more often reported a perception that those around them were improving their toilets.

**Table 6:** Agreement with sanitation related statements among respondents

Item	Control		Intervention		PD, %	95% CI	APD%	95% CI
	N	%	N	%				
Most people around here use a toilet regularly.	1214	83.6	1278	89.5	5.2	1.0/9.4	4.6	0.5/8.7
Everyone in my household uses a toilet.	1214	87.0	1278	90.9	3.8	−1.0/ 8.6	2.8	− 1.9/7.5
Many people around here are improving their toilets.	1214	71.6	1278	77.2	5.5	1.1/ 9.9	5.6	1.2/10.1
Using a toilet saves time and effort compared to open defecation.	1214	97.9	1278	98.2	0.3	−1.0/1.6	0.3	−1.0/1.6
Using a toilet builds your reputation in the community.	1214	97.6	1278	97.9	0.2	−1.2/1.7	0.0	−1.5/1.5
A smart person is one who uses a toilet.	1214	53.0	1278	51.6	−1.2	−6.5/4.2	− 1.1	−6.4/4.3
It is possible to feel proud of one’s toilet.	1214	94.9	1278	96.6	1.7	−0.2/3.5	1.4	−0.4/3.2
Most people around here think it’s good to use a toilet.	1214	96.1	1278	96.9	0.7	−1.3/2.8	0.5	− 1.6/2.6
Using a latrine gives me a ‘packed’ (claustrophobic) feeling.	1214	6.7	1278	5.5	−1.3	−3.3/0.7	−0.8	−2.8/1.1
Toilets are not just for women; men should use them too.	1214	81.0	1278	79.4	−1.2	−6.3/4.0	− 1.0	−6.2/4.2
It is appropriate to have a toilet as good as your house.	1214	98.4	1278	98.6	0.1	−0.9/1.2	0.0	−1.0/1.1
It is ok for poor people to practice open defecation.	1214	21.6	1278	17.7	−4.0	−7.1/−1.0	−3.3	−6.2/−0.4
Toilet pits fill quickly if too many people in the household use them.	1214	66.1	1278	65.7	0.1	−4.7/5.0	0.5	−4.2/5.3
Most of the people I care about think I should use a toilet.	1214	96.0	1278	95.9	−0.1	−1.9/1.7	−0.2	−1.9/1.4
People around here think a household should have a good toilet.	1214	97.9	1278	98.0	0.1	−1.1/1.3	0.1	−1.1/1.3
Even if no one else around here had a good toilet, I would still make sure I had one.	1214	91.4	1278	94.1	2.7	−0.4/5.8	2.1	−0.9/5.2
During farming season, most people around here defecate in the field/open	1214	68.7	1278	62.5	−5.9	−11.0/0.8	−4.9	−9.8/0.1
Defecating in the field is more convenient than using a toilet	1214	18.4	1278	17.8	−0.5	−4.1/3.2	0.4	−3.3/4.1
Having a good toilet at home is a mark of better status in the village	1214	98.1	1278	98.1	−0.1	−1.3/1.2	−0.1	−1.3/1.1

*PD* prevalence difference, calculated using linear regression (function: Gaussian, link: identity). Clustering at village level was adjusted for by using generalised estimating equations and robust standard errors. *APD* adjusted prevalence difference. PD was adjusted for asset index (continuous variable) and maximum male education level (dichotomised into primary or less vs secondary or higher)

**Fig. 3:**
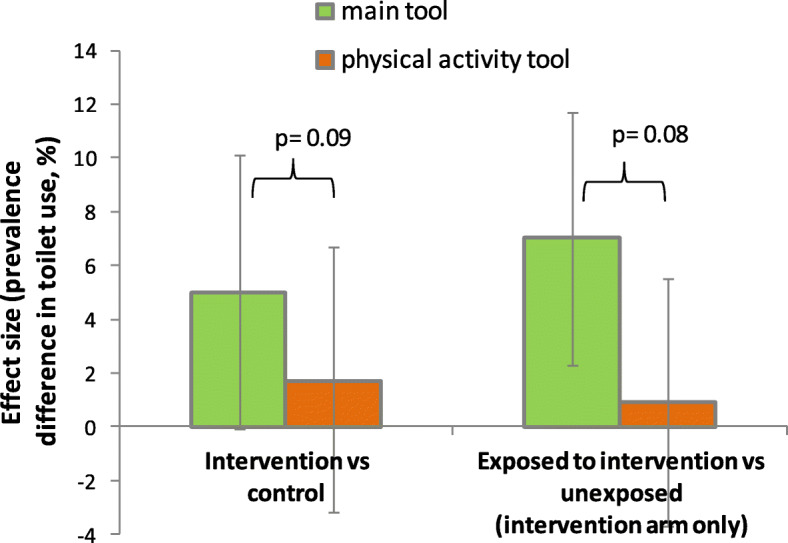
Comparison of effect sizes resulting from the two questionnaire tools used in the study. The left side shows the effect size of the intervention on toilet use vs control (+ 5.0% for the main tool, + 1.7% for the physical activity tool). The right side shows the effect sizes on toilet use among participants exposed to the intervention vs those not exposed in the intervention arm (+ 7.0% for the main tool, + 0.9% for the physical activity tool). *P* values denote test for interaction

In the intervention arm, reported use of toilet by all household members (main questionnaire tool) was 96.1% in those reporting to have heard of the 5 star campaign, and 89.1% in those that had not heard of it (prevalence difference + 7.0%, 95%CI 2.2% / 11.7%). By contrast, again focussing on the intervention arm, there was no difference in reported toilet use measured by the physical activity questionnaire between exposed and unexposed participants (86.8% vs 85.9%, prevalence difference 0.9%, 95%CI -3.7% / 5.5%). To explore the statistical support for this difference in the effect of campaign exposure on reported toilet use between the two tools, we conducted a test for interaction between type of questionnaire (main tool vs physical activity tool) and having heard of the campaign in the intervention arm which showed a *p* value of 0.08 (Fig. 3). This provides some support for the idea that in the intervention arm over-reporting of the outcome (reported toilet use) occurred especially in those exposed to the campaign, while the physical activity tool minimised over-reporting related to campaign exposure.

### Process evaluation

The process evaluation revealed several challenges in the delivery of the intervention. Most prominently, the scattered population made it difficult to reach all households within the time and budget available. Often, due to hot weather, people did not want to step out of their homes in the afternoon to participate in the street events. Ongoing agricultural work or other livelihood activities meant that many people either lived on the farmland or did not return home until around late in the evening. Some communities did not encourage women to participate in evening events. We found that the role of the volunteers remained weak as the implementation team could not recruit volunteers in 10 clusters, and in some clusters their engagement could not be sustained beyond the Day 1 event. During field observations (*n* = 6) and exit interviews (*n* = 6) with participants after the Day 1 and Day 2 events, we found that participants had largely understood the campaign messages. Not all participants in the evening events were exposed to the campaign materials such as the toilet model, world of toilets and virtual reality film showcased during the street events. In the evening event where certificates were awarded to those with 5 star toilets, participants in large numbers expressed the desire to get their toilets five-star certified. The skit was the most recalled event of Day 1. Participants’ recollections of Day 2 event activities included the process of how faeces converts into compost, the mad scientist video on reducing pit filling anxiety, and the board with pictures of families with a 5 star toilet.

The digital elements of the intervention were on the whole well received and generated a lot of interest, but were hard to deliver at scale. For example, the Virtual Reality to showcase the 5 star toilet attracted crowds, but was limited by how many can view it at any point.

## Discussion

The intervention was associated with a 5% point increase in households that reported toilet use by all members aged 5 years or above. In the control arm, about 85% of households were consistent toilet users. In this light, a 5% increase could be interpreted as a relevant effect given that only around 15% of the target households (households with an existing toilet) may have been inconsistent or non-users prior to intervention. It could therefore be argued that a third of those households who were in a position to improve toilet use behaviour, did so as a consequence of the campaign. However, our alternative tool to measure toilet use, the physical activity questionnaire, which we assume to be less likely to be influenced by responder bias, showed no evidence for an effect. We believe the effect estimate of the primary outcome is likely to be subject to over-reporting of toilet use in the intervention arm. This is supported by the comparison of exposed and unexposed in the intervention arm, which resulted in a major difference in the primary outcome measured by the main tool, but not in the physical activity tool (summarised in Fig. 3). The results therefore suggest that the 5 Star Toilet campaign in this rural Indian setting with high pre-existing toilet coverage and probable high levels of use did not further increase toilet use.

The campaign was delivered by trained facilitators and follow-up in the community was done through village volunteers. The word ‘smart’ was translated as *saru (good), saras (nice) and sunder (beautiful)* by the facilitators while delivering the campaign. In this manner the campaign attempted to mainstream the 5 Star Toilet concept by placing it in the context of other desirable, modern things in people’s lives.

There are a number of possible reasons for the failure of the intervention to achieve major changes in toilet use behaviour. First the intervention may have been ill conceived, second it may have failed to reach enough of the target audience with enough intensity to effect measurable change, third it may have been delivered to a population who were already convinced of the need to use toilets, leaving only a small number of potential users who could not be persuaded.

Process evaluation data suggests that, for those who participated, the programme was well received. Through interviews and focus group discussions with participants exposed to the campaign and regional government representatives, we found that intervention components surprised the participants and were different from what people may have experienced before, in government or NGO-led initiatives. Through discussions with participants, the most commonly reported motives for toilet improvement included comfort, convenience, affiliation, status and honour related to women’s safety. It thus appeared that our theory of change for how the intervention would lead to toilet use was supported, at least for those who received the intervention.

However, the exposure of the target population to the intervention was low. Only about 10–15% of the intervention households showed evidence of exposure to the intervention. This low exposure was insufficient to change the study population’s perceptions around toilet ownership and other relevant sanitation-related factors at village level. Small positive changes in toilet features and proxy markers of current use were observed (see Table 4) but statistical support for these changes was low and could have occurred by chance.

The low exposure to the 5 Star Toilet campaign may have been due to the fact that clusters in the study area were geographically spread out and various socio-economic strata and caste groups/religious communities lived in different segments of the village. This challenged the campaign facilitators as within the short timeframe available for the day event, it was difficult to reach out to each and every household. The main occupation of people in the study clusters was agricultural, managing livestock and diamond polishing, and many had also migrated to the nearby cities of Surat and Ahmadabad. The absence of householders made it difficult for the campaign facilitators to identify eligible households and to recruit participants for the intervention. It is also plausible that the non-users of toilets were a particularly hard-to contact and hard-to-convince group, since many of those around them had already adopted the practice.

On the whole, intervention intensity was temporarily high in the locations where activities occurred but geographically too scattered and too infrequent to achieve a high exposure at population level. The digital elements of the interventions were well received by the population, but were found to be no substitute for the sustained efforts on the ground likely to be required to achieve behaviour change.

The study findings are in line with other water/sanitation/hygiene-related behaviour change campaigns, such as those targeting handwashing behaviour where small interventions have shown success [17], while larger campaigns at scale have failed to produce major effects [23–25]. Delivering behaviour change interventions at scale remains a challenge. Three other trials conducted under the same initiative alongside the present study in other parts of India also failed to achieve relevant changes in toilet use, even though these were carried out in different settings with lower toilet coverage and possibly lower baseline toilet use [20].

Rates of usage of toilets, at 84–88%, by a variety of reported measures, were higher in this study than we initially expected, based on small scale surveys prior to the intervention. One solution to the problem of intervening in a population who in the majority did not need to change behaviour might have been to find a way to target only the approximately 15% of people or households who were not using their latrines consistently. From the programme perspective, intervention efficiency will be reduced if it mainly consists of activities performed at the community level such as public events and road shows. Intervention resources are then wasted on a majority of people attending such events who have no need to change their behaviour. On the other hand, identifying target households within a given community may not be easy without in-depth knowledge from inside the community and serial household visits to increase intervention exposure in those who could benefit from it most. Approaches to identify households not using toilets need to be conducted in a way that avoids stigmatising households based on income, caste and other status-related characteristics. Thorough formative research taking into account knowledge from earlier programmes will be needed to guide the decision on whether to favour a community-level or more targeted approach for a given intervention in a particular setting.

Limitations of the present study include the use of self-reported behaviour to measure the primary outcome, imbalances in some socio-economic variables across study arms, the low coverage of the intervention and the short time frame from randomisation to intervention delivery and outcome assessment.

The study relied on self- or proxy-reported toilet use as the primary outcome, which is likely to lead to over-reporting of socially desirable behaviours. This method was used across all four studies in this programme to ensure standardised reporting of the primary outcome. In the setting of a randomised trial testing the effect of an intervention on socially desirable behaviours (here: toilet use), there is an additional risk of differential reporting behaviour between intervention and control arm. Study participants in the intervention arm who have just been exposed to an intervention may be more prone to over-reporting toilet use than participants in the control arm, for whom the survey may simply appear as just another household survey, unlinked to an intervention. Higher over-reporting of toilet use in the intervention would cause a spurious effect of the intervention on toilet use.

We tried to explore the potential for differential over-reporting influencing the study results by employing a newly developed tool to measure toilet use and open defecation – the physical activity tool. Here, going for open defecation was one of many questionnaire items related to different physical activities throughout the day, alongside other questions related to chronic non-communicable diseases including dietary pattern. This tool found a 4.4% points lower toilet use among all study participants (individual level), and there was no evidence for an increase in toilet use among the intervention households. These findings are compatible with the presence of over-reporting in the primary outcome, and suggest that the observed effect of a 5% percentage point increase in toilet use may be due to differential over-reporting. This is supported by the conspicuous difference in reported toilet use in the intervention arm between those reporting to have heard of the campaign and those that had not. The effect of exposure on reported toilet use clearly depended on the questionnaire (Fig. 3). The main questionnaire showed higher reported toilet use among those in the intervention arm directly exposed to the campaign compared to the unexposed. By contrast, the physical activity tool showed no difference in this comparison. In our view, this is evidence for campaign exposure changing reporting behaviour but not toilet use. The difference between the two tools in the control arm was not great (84% vs 81%, Table 3), suggesting that in the absence of an intervention, and if households are visited only once, over-reporting may be limited.

**Fig. 4:**
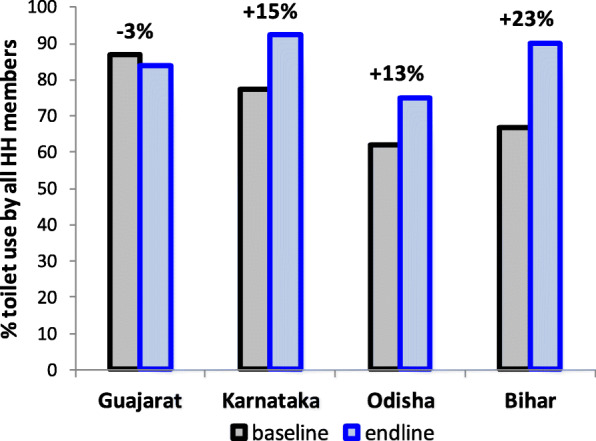
Comparison of baseline to endline change in reported toilet use in control arm across all 4 trials participating in the programme, adapted from [20]. The columns for Gujarat describe the findings from the present study

Ahead of the study we suspected over-reporting to occur even in the control arm if households are visited repeatedly before and after the intervention. We tried to reduce this potential for over-reporting by not repeating questions related to sanitation and toilet use in the same households at baseline and at follow up. Households undergoing these questions at baseline were discarded from further study. This strategy appears to have been successful. Toilet use by all household members at baseline (overall 85%, 87% in the control arm, 83% in the intervention arm) was similar to toilet use by all household members observed in the control arm at follow up (84%), suggesting that the trial procedures did not influence reporting behaviour. These findings further suggest that administration of the physical activity tool, which was done about 7 to 10 days before the endline tool, did not influence responses of the endline tool, possibly by successfully camouflaging the purpose of the physical activity survey as a health and lifestyle survey. The lack of increase in reported toilet use from baseline to follow up is in contrast to findings from the other three trials which were part of this initiative. These trials employed similar study and intervention designs, targeting the same health behaviour (consistent use of already built toilets by all household members). They all used a similar tool to measure the primary outcome of reported toilet use behaviour at individual level in each household. However, these trials revisited the same households at baseline and follow up, whereas we removed all households in the baseline toilet use study from further study. Unlike in our study, these trials found a marked increase in reported toilet use in the control arm (Fig. 4, data extracted from [20]). One could argue that it may have been less straightforward for households in our study to link the purpose of the questionnaire to the intervention compared to studies repeating the same questionnaire in the same people with an intervention in between. However, the strong increase in reported toilet use found in the control arm in the other three trials may also have been due to an increase in state- or district-level sanitation activities immediately following the baseline, thus contaminating the trial sites [20].

The lack of baseline toilet use data in the households included in the endline survey meant that we could not adjust the effect estimates for any imbalances in the primary outcome, or use such data for restricted randomisation to achieve balance. Some endline imbalances were observed in variables associated with the primary outcome (asset index and male education). Adjusting for these variables attenuated the observed effect sizes, but did not fundamentally change the interpretation of the results. On the whole we believe that minimising over-reporting and bias by not revisiting the same households before and after an intervention is more important than achieving a high degree of baseline balance across arms with regard to the study outcome. Bias is difficult to address analytically whereas imbalances are due to a chance process which can be adjusted for (at least to some extent) and interpreted in the light of confidence intervals and the results of other studies (in meta-analysis).

Finally, for programmatic reasons, the trial had to be conducted in a short time frame. Less than 12 months were available for baseline study, randomisation, intervention delivery, outcome assessment and reporting of results. Due to budget constraints, intervention activities in each village had to be reduced to 2 days in total. In retrospect, we doubt whether interventions requiring households to make changes to their toilets and to motivate household members to change their toilet use behaviours can result in success within such a short timeframe and with such limited resources.

## Conclusion

The 5 star toilet intervention did not achieve a convincing increase in toilet use behaviour in this rural Indian setting with high toilet coverage and high usage. Insufficient geographic spread within clusters and low exposure among those most likely to benefit from the campaign are likely to have contributed to the low impact of the intervention. Interventions only working at community level without visits to individual households may be relatively cheap and scalable but may become inefficient if only a minority of the population are potential beneficiaries. Future research could be directed to how to better target large scale sanitation interventions to sub-populations at greatest need, without stigmatising economically and socially disadvantaged groups. It further needs to be established how large scale sanitation campaigns can be incorporated into the overall sanitation strategy at local, district, state and national level.

Researchers need to develop better tools for assessing toilet use that are not prone to over-reporting and, in particular, differential over-reporting between an intervention and a control arm. Trials should not rely on self-reported toilet use as the only method for outcome assessment if the questionnaire design makes the purpose of the study obvious to study participants.

## Supplementary information

**Additional file 1.** Physical activity questionnaire.

## Data Availability

The datasets generated and/or analysed during the current study are available from the authors on request.

## Abbreviations

BCD: Behaviour Centred Design
CI: Confidence interval
ICC: Intra-class correlation coefficient
RANAS: Risks, Attitudes, Norms, Abilities, and Self-regulation framework
SBM-G: Swacch Bharat Mission Gramin
ToC: Theory of Change
