# Supplementary material for: Cluster-randomised trial to test the effect of a behaviour change intervention on toilet use in rural India: results and methodological considerations
Source: BMC Public Health. 2020 Sep 11;20:1389. doi: 10.1186/s12889-020-09501-y (PMC7488773; doi:10.1186/s12889-020-09501-y)
Supplement: Supplementary file 1 — Additional file 1. Physical activity questionnaire. [file 12889_2020_9501_MOESM1_ESM.docx]

**Physical activity questionnaire**

| **N^o^** | **Variable name** | **Question** | **Response** | **What to do** |
| --- | --- | --- | --- | --- |
| 1 | *villid* | Village identifier: ગામની ઓળખ | ___ | Write ID |
| 2 | *hhid* | Household identifier : ઘરની ઓળખ | ___ | Write ID |
|  | *name* | What is your name? તમારું નામ જણાવો | ______________________ | Write name |
| 3 | *sex* | Gender : જાતિ | 0-M 0- પુરુષ  1-F ૧ – સ્ત્રી | Circle |
|  | *age* | What is your age? તમારી ઉંમર શું છે? | ____ | Write age |
| 4 | *diab* | Are you taking tablets for diabetes? : શું તમે ડાયાબીટીસની દવા લો છો? | 0-NO 0- ના  1-YES ૧ – હા | Circle |
| 5 | *bp* | Are you taking tablets for hypertension? શું તમે બ્લડ પ્રેશરની દવા લો છો? | 0-NO  1-YES | Circle |
| 6 | *chd* | Are you taking tablets for heart disease? શું તમે હ્રદયની કોઈ દવા લો છો? | 0-NO  1-YES | Circle |
| 7 | *heigh* | What is your height? તમારી ઉંચાઇ કેટલી છે? | ___  999-don’t know | Write height in cm |
| 8 | *weigh* | What is your approximate weight? તમારૂ વજન કેટલુ છે? | ___  999-don’t know | Write weight in kg |
| 9 | *fruits* | In the last week how times did you eat fruit approximately? I will give you 4 options.  READ ALL OPTIONS  છેલ્લા અઠવાડિયા માં તમે કેટલી વાર ફળો ખાધા છે? | 1. NEVER 2. One day only 3. Most days 4. ALL days | Write down number |
| 10 | *nuts* | In the last week how times did you eat nuts approximately? I will give you 4 options.  READ ALL OPTIONS  છેલ્લા અઠવાડિયામાં તમે કેટલી વાર સુકામેવો અથવા સીગ દાણા ખાધા છે ? | 1. NEVER 2. One day only 3. Most days 4. ALL days | Circle |
| 11 | *fish* | In the last week how times did you eat non-vegetarian food approximately? I will give you 4 options.  READ ALL OPTIONS  છેલ્લા અઠવાડિયામાં તમે કેટલીવાર માંસ અથવા મચ્છી ખાધા છે? | 1. NEVER 2. One day only 3. Most days 4. ALL days | circle |
| 12 | *buy* | Yesterday how many minutes did you walk to buy things you need? I will give you 4 options.  ગઈ કાલે તમે જરૂરી વસ્તુઓ ખરીદવા માટે તમે કેટલા મિનિટ ચાલ્યા?  READ ALL OPTIONS | 1. Less than 10 minutes 2. Between 10 and 30 minutes 3. More than 30 min 4. Did NOT walk to buy things | circle |
| 13 | *rel* | Yesterday how many minutes did you walk to visit friends or relatives? I will give you 4 options.  ગઈ કાલે તમે મિત્રો અથવા સંબંધીઓને મળવા કેટલા મિનિટ ચાલ્યા?  READ ALL OPTIONS | 1. Less than 10 minutes 2. Between 10 and 30 minutes 3. More than 30 min 4. Did NOT walk to visit friends or relatives | Circle |
| 14 | *field* | Yesterday how many minutes did you work in the fields? I will give you 4 options.  ગઈ કાલે તમે ખેતરમાં કેટલા મિનિટ કામ કર્યું?  READ ALL OPTIONS | 1. Less than 30 minutes 2. Between 30 and 1 hour 3. More than 1 hour 4. Did NOT work in the field | Circle |
| 15 | *anim* | Yesterday how many minutes did you walk to take animals (cows or goats) to places where they feed? I will give you 4 options.  ગઈ કાલે તમે પ્રાણીઓને (ગાય અથવા બકરા) ચરાવવા કેટલા મિનિટ ચાલ્યા?  READ ALL OPTIONS | 1. Less than 10 minutes 2. Between 10 and 30 minutes 3. More than 30 min 4. Did NOT walk to take animals around | Circle |
| 16 | *wat* | Yesterday how many minutes did you walk to fetch water? I will give you 4 options.  ગઈ કાલે તમે પાણી લાવવા માટે કેટલા મિનિટ ચાલ્યા?  READ ALL OPTIONS | 1. Less than 10 minutes 2. Between 10 and 30 minutes 3. More than 30 min 4. Did NOT walk to fetch water | Circle |
| 17 | *def* | Last time you defecated how many minutes did you walk to go to the fields for defecation? I will give you 4 options.  છેલ્લે જ્યારે તમે ખુલ્લામાં સંડાસ જવા માટે ગયા ત્યારે તમે કેટલા મિનિટ ચાલ્યા?  READ ALL OPTIONS | 1. Less than 10 minutes 2. Between 10 and 30 minutes 3. More than 30 min 4. Used toilet at home | Circle |
| 18 | *work* | Yesterday how many minutes did you walk to place of work? I will give you 4 options.  ગઈ કાલે તમે કામના સ્થળે જવા માટે કેટલા મિનિટ ચાલ્યા?  READ ALL OPTIONS | 1. Less than 10 minutes 2. Between 10 and 30 minutes 3. More than 30 min 4. Did not walk to work | Circle |
| 19 | *leis* | Yesterday how many minutes did you walk for fun/relaxation? I will give you 4 options.  ગઈ કાલે તમે આનંદ / આરામ માટે કેટલા મિનિટ ચાલ્યા?  READ ALL OPTIONS | 1. Less than 10 minutes 2. Between 10 and 30 minutes 3. More than 30 min 4. Did not walk for fun / relaxation | Circle |
| 20 | *other* | Yesterday how many minutes did you walk for other purposes? I will give you 4 options.  ગઈ કાલે અન્ય કાર્ય માટે તમે કેટલા મિનિટ ચાલ્યા?  READ ALL OPTIONS | 1. Less than 10 minutes 2. Between 10 and 30 minutes 3. More than 30 min   4.Did not walk for other purposes | Circle |
|  | | | | |
